# Cross-cultural adaptation of instruments assessing breastfeeding determinants: a multi-step approach

**DOI:** 10.1186/1746-4358-9-16

**Published:** 2014-09-21

**Authors:** Emily L Tuthill, Lisa M Butler, Jacqueline M McGrath, Regina M Cusson, Gracia Nokhaya Makiwane, Robert K Gable, Jeffrey D Fisher

**Affiliations:** 1 University of Connecticut School of Nursing, 231 Glenbrook Rd, Storrs, CT 06269, USA; 2 Center for Health Intervention and Prevention, University of Connecticut, 2006 Hillside Rd, Unit 1248, Storrs, CT 06269, USA; 3 Department of Medicine, Division of General Pediatrics, Boston Children’s Hospital, Boston, MA 02215, USA; 4 Department of Pediatrics, Harvard Medical School, Boston, MA 02215, USA; 5 Connecticut Children’s Medical Center, Institute for Nursing Research and Evidence-Based Practice, 282 Washington St., Hartford, CT 06106, USA; 6 University of KwaZulu-Natal, King Edward Avenue, Scottsville, Pietermaritzburg, South Africa; 7 Johnson & Wales University, Center for Research and Evaluation, 8 Abbott Park Place, Providence, RI 02903, USA; 8 College of Arts & Sciences, Johnson & Wales University, 8 Abbott Park Place, Providence, RI 02903, USA

**Keywords:** Breastfeeding, Cross-cultural adaptation, Translation, Scale development, Content validity, Breastfeeding scales

## Abstract

**Background:**

Cross-cultural adaptation is a necessary process to effectively use existing instruments in other cultural and language settings. The process of cross-culturally adapting, including translation, of existing instruments is considered a critical set to establishing a meaningful instrument for use in another setting. Using a multi-step approach is considered best practice in achieving cultural and semantic equivalence of the adapted version. We aimed to ensure the content validity of our instruments in the cultural context of KwaZulu-Natal, South Africa.

**Methods:**

The Iowa Infant Feeding Attitudes Scale, Breastfeeding Self-Efficacy Scale-Short Form and additional items comprise our consolidated instrument, which was cross-culturally adapted utilizing a multi-step approach during August 2012. Cross-cultural adaptation was achieved through steps to maintain content validity and attain semantic equivalence in the target version. Specifically, Lynn’s recommendation to apply an item-level content validity index score was followed. The revised instrument was translated and back-translated. To ensure semantic equivalence, Brislin’s back-translation approach was utilized followed by the committee review to address any discrepancies that emerged from translation.

**Results:**

Our consolidated instrument was adapted to be culturally relevant and translated to yield more reliable and valid results for use in our larger research study to measure infant feeding determinants effectively in our target cultural context.

**Conclusions:**

Undertaking rigorous steps to effectively ensure cross-cultural adaptation increases our confidence that the conclusions we make based on our self-report instrument(s) will be stronger. In this way, our aim to achieve strong cross-cultural adaptation of our consolidated instruments was achieved while also providing a clear framework for other researchers choosing to utilize existing instruments for work in other cultural, geographic and population settings.

## Background

Cross-cultural adaptation is an approach taken to utilize existing instruments in other cultural, language or geographic settings. There are multiple advantages to adapting an existing instrument for a study, including cost savings, time savings, and the relatively fewer steps required for effective instrument adaptation compared to developing a new instrument [1]. In addition, a well-developed instrument with robust validity and reliability of the data in the source version rigorously adapted and translated into several languages allows for international studies to compare results of specific constructs across cultures and languages. Thus, a field that utilizes existing instruments can build a knowledge base in which generalizations can be made and discussed across cultures in efforts to impact global public health. However, for an instrument to be used outside the original setting (i.e., source language and cultural context), cultural adaptation and translation are needed [2].

Cross-cultural adaptation is defined as a process that looks at both language (i.e., translation) and cultural adaptation (i.e., culturally relevant content) for use in another setting [2]. Today an increasing body of literature across disciplines [1-4] describes achieving effective cross-cultural adaptation by following multiple validation steps. The most common of these steps include content validation utilizing content expert feedback, translation and back-translation [2]. Thus, through the use of multiple recognized methods, rigorously followed, achieving a culturally equivalent instrument can result. In addition to using established, commonly followed approaches, we also applied a content validity index score (described below and in Additional file 1), which provides a more standardized approach to decision-making based on quantitative input from content experts to increase the strength of our content validity. This article explores cross-cultural adaptation and the process taken to prepare our instrument, which is a compilation of (1) the Iowa Infant Feeding Attitudes Scale (IIFAS), (2) the Breastfeeding Self-efficacy Scale-Short Form (BSES-SF) and (3) additional newly developed items, for use in another setting.

### Rationale for cross-cultural adaptation

The purpose of adapting and translating the IIFAS and BSES-SF is for use in an intervention study to increase exclusive breastfeeding in KwaZulu-Natal, South Africa. Grounded in behavioral change theory and using the Information, Motivation and Behavioral Skills (IMB) model [5] as our conceptual framework, we will develop a brief intervention that enhances IMB breastfeeding determinants to support HIV-positive mothers to exclusively breastfeed in a region suffering from the highest HIV burden worldwide [6].

The decision to adapt and translate the IIFAS and BSES-SF rather than create a new instrument was based on a number of considerations. First, both instruments have been widely used in infant feeding studies to measure our desired constructs (i.e., infant feeding information, motivation, and behavioral skills) with results demonstrating strong validity and reliability of the data [7,8]. This being said, use of the IIFAS and BSES-SF still required additional items to be developed in order to fully capture our three constructs (explained in further detail below). Second, these measures have successfully been translated and used in other cultures apart from their original target population [8]. Third, given the timeframe and resources of the overall study, the choice to adapt existing instruments provided increased project feasibility. Finally, despite KwaZulu-Natal accounting for the highest prevalence of HIV infections worldwide [9] and several quantitative studies [10-12] conducted there, a published infant feeding instrument assessing mothers’ affective orientation toward infant feeding behavior in isiZulu is lacking. Although dissemination of instrument adaptation and translation from English into isiZulu has occurred in the literature [13], this process has not occurred for a breastfeeding instrument. Therefore, our cultural adaptation and translation process sought to achieve cross-cultural equivalency while increasing infant feeding data by using existing instruments. In addition, we believe this detailed process can serve as a resource for other researchers conducting cross-cultural adaptation of existing instruments.

### Existing instruments

Permission was obtained in August, 2012 from Drs. De La Mora and Dennis who originally designed and who hold copyright of the IIFAS and the BSES-SF, respectively. The following details the original psychometric properties of each instrument.

### IIFAS instrument

The IIFAS was developed by Drs. De la Mora and Russell [14] to measure information and attitudes toward infant feeding. They tested their instrument in multiple studies resulting in adequate predictive validity and internal consistency ranging from a Cronbach’s alpha of 0.79 to 0.86 [14,15]. The scale consists of 17 items with a 5-point Likert-type scale with scores ranging from 1 *(strongly disagree)* to 5 *(strongly agree)* that can predict the choice of infant-feeding method as reflected by measures of behavioral intentions, as well as the individual’s actual feeding behavior and the duration of the behavior. In addition, other researchers have used the instrument to measure infant feeding information and attitudes successfully [8].

### BSES-SF instrument

The BSES-SF is a refined version of the larger BSES (33-items) and includes 14 items. The BSES-SF was developed to measure mothers’ breastfeeding self-efficacy (i.e., their perceived ability to perform breastfeeding). The BSES-SF has been tested among breastfeeding mothers with data ranging in Cronbach alpha scores from 0.90 to 0.94 [8]. Dennis [16] reworked the instrument into its current form, which she renamed as the BSES-Short Form (BSES-SF). The instrument uses a 5-point Likert Scale with 1 *(not at all confident)* and 5 *(always confident)*. Predictive validity was seen with higher scores on the BSES-SF at 1-week postpartum linked to the mother being more likely to be breastfeeding at 4 and 8 weeks postpartum and doing so exclusively. The BSES-SF has been successfully adapted into Chinese [17] with reported strong psychometric results [13].

### Maintaining content validity in cross-cultural adaptation

Content validity is the extent to which the items reflect from the intended universe of content [18]. In other words, strong content validity represents items being both relevant and representative of the phenomenon under study [19]. Evidence of content validity is derived from both literature review and expert judges [20]. An instrument lacking content validity results in poor reliability of the data and a tool that fails to measure its intended constructs [21]. In order to achieve cross-cultural equivalency (i.e., maintain content validity for use in different cultural setting), items must be culturally adapted [2]. Applying a content validity index score provides a more objective approach to testing content validity (see Additional file 1).

There are several options when choosing to apply a CVI. One valid approach is to calculate the average congruency percentage [22] as seen in Beck & Gable [21]. In this method, experts are asked to determine the congruence of each item with the researcher’s original domain specifications. The items are then converted to a percentage. A total mean percentage for all experts is then calculated for each item. A score of 90% or higher is considered adequate [21]. Another equally effective approach is to apply the CVI score. This can be done as a scale level CVI (s-CVI), which is calculated either through universal agreement among experts or as an average of item-level CVIs [20]. However, individual item indices have been shown more valid than a scale score expressing overall content validity of the instrument [20]. Applying rigorous efforts to ensure content validity is considered particularly important given that content validity is the most subjective validity to measure and the degree to which an instrument has achieved content validity can be difficult to assess [21].

For the purpose of this project, content validity for cross-cultural equivalency was achieved through consultation with expert judges to yield an item-level CVI score [19]. Six content experts were all stakeholders in the field of mother-to-child transmission of HIV, breastfeeding and/or infant feeding in South Africa. Specifically, three experts were researchers at the University of KwaZulu-Natal, one a researcher at a non-governmental organization working in infant feeding and HIV, one expert was a US researcher working in South Africa with HIV-positive women and infant feeding, and one was a healthcare provider.

### Translation

Brislin’s approach was undertaken to back-translation to determine semantic equivalence in the translated version [6]. Although some researchers suggest translating the instrument prior to cultural content adaptation [2], this was not appropriate given the fact that content experts within the field of infant feeding conducting research in KwaZulu-Natal (i.e., the target setting) are English speaking only. Therefore, to ensure content validity and cultural relevance and to avoid multiple translation efforts, our content experts reviewed our instrument and made recommendations on the English version prior to translation. By following these two established methodologies [6,19], the process to reach cross-cultural equivalency as defined by Beaton et al. was met [2]. Similar procedures have been documented and well illustrated and have served as a guide [6-8]. Given the conclusions we make based on self-reported answers to instrument items, the importance of rigorous cross-cultural adaptation is a critical step in our overall study design.

## Methods

### Additional item development

Additional item development was needed for two reasons: first, to fully capture our motivation construct and second to ensure items spanned infant feeding content in the context of HIV. Specifically, the IIFAS and BSES-SF focus on infant feeding information, attitudes and self-efficacy. However, by only assessing attitudes they fail to adequately measure motivation, which is comprised of attitudes and social normative support [5]. Furthermore, attitudes and social normative support in the context of HIV are unique (e.g., perceptions of community support for exclusive breastfeeding, stigma) and require a modified approach over items targeting non HIV-positive women. Therefore, social norm items in the context of HIV were developed following item development techniques [18].

The development stage involved applying our conceptual/operational definition and review of the literature to create our items [18]. The conceptual definition of our motivational construct states “an HIV-positive woman’s beliefs toward infant feeding and her perceived level of support and cultural traditions toward infant feeding will determine her desire (motivation) to practice a specific infant feeding behavior”. To generate these new items, findings from existing qualitative studies [23] surrounding social normative support in the context of HIV and infant feeding were applied. Themes and quotes reported in qualitative work highlighted barriers, such as stigma, disclosure and traditional norms, which informed the content of new items [23]. Discussions with healthcare providers and mothers with direct infant feeding experience in the context of HIV also occurred to help preserve meaning of expression and content for that item. Increasing understanding from those with direct experience regarding a construct affords depth and a connection to reality that the literature alone may not elucidate [21]. To enhance our potential inter-rater reliability we matched the number of social norm items with the number of attitude items. Therefore, a total of 16 items were generated; 13 items to complete the motivation construct of our instrument thus matching the 10 attitude items found in the IIFAS and 3 items to measure self-efficacy in identifying breast health issues in the context of HIV. The newly developed items were then added to our consolidated instrument (i.e., IIFAS, BSES-SF) to reach a total of 47 items.

### Adaptation of the IIFAS

The IIFAS is spilt directionally with half of the items favorable to breastfeeding and half favorable to formula feeding. Multiple directions may elicit more honest responses from respondents because they must read each item carefully to make their decision. However, multiple directions can also cause confusion and is not recommended by some instrument developers [18]. Therefore, items were adapted to be positive in direction. This decision was made in consultation with an expert in instrument development and content experts in the US. Making all items one-directional simplified the instrument’s readability making it more understandable and likely more effective in a low-literacy setting. At this point we proceeded to testing content validity for our culturally specific setting by eliciting feedback from content experts.

### Obtaining judgmental evidence through content experts

To ensure a *posteriori* content validity of our existing and newly developed items within the cultural context of KwaZulu-Natal, South Africa, the following steps outlined by Lynn and undertaken by Beck and Gable and Schilling et al., were followed [7,18,21]. Similar to the committee approach (where a group comes together to discuss the items in detail and to resolve any concerns in cultural relevance or semantics with the rationale that an issue missed by one can be caught by another [6]), content experts were utilized to assess the content validity of each item through a quantifying application [19]. Content experts are expected to be both experienced in the field being investigated and familiar with the literature on the topic of interest [19]. The number of content experts needed is somewhat arbitrary [19]. However, there are some established rules on choosing this number, including recommending a minimum of three content experts, but more than 10 is probably unnecessary [19].

The item- level CVI (I-CVI) was applied in the herein study and is determined after each content expert rates the item on its relevance. Relevance is defined by its fit, understandability and overall clarity using a 4-point Likert scale [19]. The I-CVI is computed based on the number of experts who gave the item a score of 3 or 4 (i.e., relevant or highly relevant, and thus dichotomizing the items to either relevant or not relevant) divided by the total number of experts. Keeping in mind the possibility of agreement occurring by chance alone criteria for item scoring based on the number of content experts was developed [19]. Therefore, in a panel with five or fewer judges, all must agree on the content validity rating for the item to be considered a reasonable representation of all possible ratings (i.e., I-CVI should equal 1) [19]. When there are six or more judges the standard can be relaxed, but Lynn [19] recommends no lower than .78 (e.g., with six judges there could be one rating of “not relevant” (I-CVI = 0.83)). This recommendation is useful to guide researchers in applying standardized rules to item revision, elimination, or suitable without revisions.

### Process for obtaining I-CVI score

Six content experts were recruited through the collaboration of the PI’s onsite mentor and study collaborators. Content experts rated the 47 item compiled instrument for its clarity, relevance, and fit using a four-point Likert content validity scale (i.e. 1-*unclear*, 2-*unable to assess without major revision of the item*, 3-*relevant with minor revision*, 4-*relevant-no changes needed*). After the content experts completed their review, the PI met in person with three of the six content experts to further discuss items that required revisions.

### Results of content validity index scoring

To obtain an I-CVI each item was scored using a cut-off of .78 or lower. Thus, each item’s rating of 3 or 4 on the Likert scale (described above) was calculated and then divided by the total number of content experts (i.e., 6) to arrive at the I-CVI score (Additional file 1). Two of the 47 items did not meet the 0.78 cut-off. One item was eliminated from the instrument and the other was revised due to content expert feedback (see Table 1). The PI with her on-site collaborator and content experts reviewed the remaining items to revise them as needed (see Table 1). Fifteen items received an I-CVI of .83 meaning 5 of the 6 content experts gave the item a score of 3 or 4 and one a 2. Of these 15 items, each was reviewed and 7 were revised per content expert suggestions. Some of the changes included:

**Table 1:** Example item-CVI scores for retained, revised and eliminated items

**Sample item**	**Item-level CVI (n = 6)**	**Rationale/Revised item, if applicable**
**Retained items**		
Formula fed babies are more likely to be overfed than exclusively breast fed babies (IIF3)	1	Relevance of question undisputed among experts
I can always ensure that my baby is properly latched on for the whole feeding (BSE4)	1	Relevance of question undisputed among experts
**Revised items**		
Breast milk is lacking in iron (IIF2).	0.66	This question was problematic among the content experts with four calling it highly relevant and two suggesting revision, not only due to the complexity of knowledge required but also the directionality of the stem. Given the complexity of knowledge within the question and the social context the question was revised to; “Breast milk is higher in nutrients than formula”. Despite this item scoring less than 0.83 content experts felt it was an important information item to include if the wording of the item was simplified.
I can always identify signs of mastitis and yeast or breast health problems (BSE16)	0.83	This item contained multiple breast health issues that were identified as most likely being unknown to our target population. was reworded; “I can always identify signs of breast health problems”.
**Eliminated items**		
Because I am worried that other people might realize that I am HIV + if they notice me exclusively formula feeding or exclusively breastfeeding I do not feel confident practicing either choice (SN5)	0	The meaning behind this item was lost in its complexity and the content experts all suggested eliminating.

1. Revise: “Formula feeding is more convenient for me than exclusive breastfeeding” to read “Feeding my baby with formula is more convenient for me than exclusive breastfeeding” (IIF13).

2. Revise: “For me women should not breastfeed in public places such as restaurants” to read “For me women should not breastfeed in public places such as markets” (IIF17)

3. Change: “I can always identify signs of mastitis and yeast or breast health problems” to “I can always identify signs of breast health problems” (BSE16)

Most revisions consisted of word changes to simplify the statement or to use a word that was more culturally relevant (e.g., cultural-based word change from “restaurant” to “market”). Several revisions required more extensive rephrasing to fit the cultural context. One example involves an item (IIF2) that received I-CVI = 0.66. In most cases this item would be eliminated, however, in discussing the item with the content experts a consensus was reached that the stem of this item if rephrased would provide important data for our information construct. Re-phrasing of this item is described in Table 1. Eight of the 14 items that received a score of 0.83 were left unchanged. Of the items left unchanged with I-CVI = 0.83, each was discussed with the content experts on how to address the problematic component of the item and consensus was met that the item was most relevant unchanged. Overall, 29 of the 47 items scored an I-CVI = 1 and were left unchanged; 14 scored an I-CVI = 0.83 and of those, 6 were revised; 1 item scored a I-CVI = 0.66 and was revised and; 1 item score an I-CVI = 0 and was eliminated. The final translated compiled instrument consisted of 46 items. At this point translation was then started.

### Cross-cultural adaptation of the language

Effectively capturing the connotative and cultural meaning of each item in the translated version was accomplished using Brislin’s back-translation approach and guided by Polit and Beck’s conceptual framework to ensure semantic equivalence [6,20]. Back translation helps to establish semantic equivalence and involves the target version of the instrument being translated back into the source language by a translator who is unfamiliar with the original wording. Semantic equivalence is met when the translated items have the same meaning as in the original [24].

### Back-translation

In cross-cultural translations a decentralized approach may be taken where modifications are made to the translated version with the goal of replacing certain culturally specific words with ones representative within the target culture [24]. Although testing the content validity and culturally adapting items was done prior to translation, additional words or phrases in isiZulu identifiable to our translators, and missed by the English speaking content experts was probable thus validating a decentralized approach. Brislin considers this step an extension of back-translation because it attempts to move from a centered word-to-word translation to one where the words chosen are more universal to the culture where the translated instrument will be used [6]. Decentering considers both the source and target versions equal, leaving both versions amendable to revision.

To support a decentralized approach and avoid any inherent problems during back-translation the following steps were taken: 1) to increase awareness of cross-cultural nuances of terms or words bilingual translators familiar with both US and Zulu culture were hired; 2) translators were given specific instructions not to try to infer meaning from the isiZulu version back into English (i.e., to consider the isiZulu version as the original); 3) translators were asked to point out words that could have multiple meanings or translations in order to clarify connotative meaning; and 4) translators were asked to identify words that are confusing or awkward in the translated version [6,25]. One advantage to using the back-translation approach is that the researcher unfamiliar with the target language can compare source language versions, which validates translator competency and provides some control regarding adaptation changes to the researcher [25].

For back translation two bilingual professional translators were hired. The translators worked independently to translate our consolidated instrument (i.e., IIFAS, BSES-SF and additional items). First, one translated the original English version into the target language (i.e., isiZulu). Then the second, who was blinded to the original version, translated that translation (i.e., isiZulu version) back into English. Being blinded to the original version provides additional validity for the researcher in seeing the original English version compared to the back-translated version [9]. In addition, blinding affords the second translator to think of the meaning in isiZulu and its equivalent in English, which helps prevent inferring meaning. Table 2 represents an excerpt of translation and back-translation by the pair of translators. All discrepancies and instances where the translator offered several word choices to represent the original English word were discussed and resolved with the translators and research team using a committee approach.

**Table 2:** Excerpt of isiZulu translation and back-translation

**Language**	**Statement**
Example 1:	
Original English	For my baby’s health, feeding some water in the first 6 months is necessary for good health.
isiZulu	Ukwenzela impilo yomntwana wami, ukumfunza amanzi ezinyangeni zokuqala eziwu 6 kuyadingenga ukwenzela impilo enhle.
Back-translation	For the baby to be healthy, it is important that I give my baby water for the first six (6) months
Example 2:	
Original English	For my baby’s health, feeding **only** formula is best in the first 6 months.
isiZulu	Ukwenzela impilo yomntwana wami, ukumfunza Ubisi lokuncela olwenziwe ngoluyimpuphu **kuphela** yikona okuhle kakhulu ezinyangeni zokuqala eziwu 6.
Back-translation	For my baby to be healthy, I must **only** formula feed for the first six (6) months.

### Committee approach

The committee approach is recommended as a second method to reach semantic equivalence [6]. In this approach, the translators and research team discussed the words, phrases and concepts that caused discrepancies. A third bilingual translator was hired to help resolve the number of word and meaning issues. One technical issue for both content experts and translators was the use of a 5-point Likert scale. It was felt that the 5 decision points would cause confusion resulting in an over emphasis of the two end points (i.e., 1 and 5). Although analysis may be limited in variability and sensitivity, a unified consensus was that more accurate responses would result from using a 3-point Likert scale with 1(*Disagree*) to 3 (*Agree*) and 1(*Not Confident*) to 3 (*Confident*). Similar response options have been used with other South African studies [26] noting the lack of identification to a 5-point scale among South African populations. The final isiZulu version was also reviewed and consensus was reached for grammar and syntax, which needs to match that of the target language and not simply be a reflection of the source language [6]. Applying a decentralized approach means the translated isiZulu version is equivalent to the original English version despite it being different (i.e., not a word by word translation), thus enhancing its strength as a cross-cultural instrument.

## Discussion

The multi-step approach described herein pulls together a set of recognized methods to effectively address Beaton et al.’s criteria for achieving cross-cultural adaptation [2]. In this way, Lynn’s recommendation to quantify an otherwise subjective process was followed to attain strong content validity that was culturally relevant [19]. Analysis of I-CVI scores and content expert written feedback was instrumental in making well-informed revisions prior to translation and back-translation and thus avoided multiple translation attempts. This process also allowed content experts to provide feedback on the additional items developed prior to translation. Given established content and construct validity are compromised when adaptations are made making fewer changes to achieve cultural adaptation was a main consideration. However, content validity can only be maintained through cultural adaptation when using existing instruments and therefore adaptation must be recognized as a necessary step [2]. Changes and revisions made to the instrument were done with careful consideration to preserve the integrity of the original items while ensuring their relevance for our target population and purpose.

To address the language component of cross-cultural adaptation, back-translation and the committee approach resulted in a more culturally targeted version. In addition, it provided an opportunity to assess translator competency and to ensure appropriate decisions were made regarding changes to items. Furthermore, the committee approach provided a forum to address such discrepancies that emerged during back-translation and complemented the back-translation process well. Electing not to perform back-translation can result in the research team being unaware of changes to items’ meaning and intention and subsequent influence on overall conclusions. Consequently, the high number of initial discrepancies between the forward and back-translated versions illustrated the complexities in translating (e.g., the decisions on meaning and word choice translators are making). Ultimately, back-translation proved an essential step in ensuring a high-quality translated instrument with the best chance to elicit accurate responses.

Given the time and resources required for instrument development, the choice to use existing instruments was appropriate. A challenge with adapting our chosen existing instruments emerged when it became clear that item development was needed. Thus, item development to address content deficits was completed following an approach guided by McCoach et al., and guided by Lynn’s recommendations for judgmental evidence [18,19]. These additional items (i.e. 14 items) may be viewed as a weakness to the instrument given psychometric testing was not performed. However, the items developed were created with the target culture and HIV context in mind and were then reviewed and revised based on content expert feedback. In this way, the rigorous process taken to develop the additional items and subsequent testing of content validity should help overcome this potential limitation. Furthermore, pretesting among the target population is recommended [2,6]. To date, our compiled instrument has been pretested among 30 pregnant and 60 postpartum women in South Africa and they were very comfortable with the nature of the items and responded without difficulty. Given the current sample size (n = 90 women), it is not feasible to conduct a factor analysis to examine the facture structure of the instrument. This analysis will be conducted at a later phase of the data gathering process. Data from pretesting will be useful for re-assessing reliability and construct validity and is currently a limitation of our consolidated instrument given additional adaptation may be needed after pretesting to obtain a meaningful result.

## Conclusions

Given the importance of exclusive breastfeeding for providing optimal nutrition for infants and better health outcomes for their mothers, increasing rates of exclusive breastfeeding is a global initiative, including among HIV-positive mothers [27]. Therefore, understanding determinants of exclusive breastfeeding is fundamental to creating programs that offer appropriate support and counseling. Breastfeeding instruments developed or adapted for use in isiZulu are lacking in the literature. Thus, our aim to adapt two existing instruments measuring critical determinants of exclusive breastfeeding, namely exclusive breast feeding-relevant information, motivation and behavioral skills, will increase our understanding of the dynamics of breastfeeding behavior *per se* and our ability to inform a culturally targeted intervention to increase exclusive breastfeeding behavior.

## Abbreviations

BSES-SF: Breastfeeding self-efficacy scale- short form; CVI: Content validity index; EBF: Exclusive breastfeeding; HIV: Human immunodeficiency virus; IIFAS: Iowa infant feeding attitudes scale; I-CVI: Item-level content validity index; IMB: Information, motivation, behavioral skills; WHO: World health organization.

## Competing interests

The authors declare that they have no competing interests.

## Authors’ contributions

ELT contributed to the design, data acquisition, analysis and interpretation and writing of the manuscript. LMB contributed to the context expert approach, instrument development and data analysis. JMM contributed to the design, manuscript development and editing. RMC contributed to the design and editing of the manuscript. RKG consulted on the data analysis and interpretation. JDF contributed to the item development, theory-based approach and data analysis. All authors read and approved the final manuscript.

## Supplementary Material

Additional file 1 **Raw data of content validity index score per item.** IIF = Iowa Information and Attitudes Scale**. SN = Social Norm Items. BSE = Breastfeeding Self-Efficacy Scale-Short Form. Click here for file
