# Supplementary material for: Cross-cultural adaptation of instruments assessing breastfeeding determinants: a multi-step approach
Source: Int Breastfeed J. 2014 Sep 21;9:16. doi: 10.1186/1746-4358-9-16 (PMC4185181; doi:10.1186/1746-4358-9-16)
Supplement: Additional file 1 — Raw data of content validity index score per item. IIF = Iowa Information and Attitudes Scale**. SN = Social Norm Items. BSE = Breastfeeding Self-Efficacy Scale-Short Form. [file 1746-4358-9-16-S1.doc]

Additional File

Appendix a. Raw Data of Content Validity Index Score per Item*

IIF=Iowa Information and Attitudes Scale**

SN= Socal Norm Items

BSE= Breastfeeding Self-Efficacy Scale-Short Form

| Content Validity Index | |  |  |  |  |
| --- | --- | --- | --- | --- | --- |
| Item | Rated 3 or 4 | CVI score | Revised | Eliminate | Comments on Revision |
| IIF1 | 5 | .83 |  |  |  |
| IIF2 | 4 | 0.66 | x |  | Important information changed, revised to state that breast milk contains more nutrients than formula in a positive direction. |
| IIF3 | 5 | 0.83 |  |  |  |
| IIF4 | 6 | 1 |  |  |  |
| IIF5 | 6 | 1 |  |  |  |
| IIF6 | 6 | 1 |  |  |  |
| IIF7 | 6 | 1 |  |  |  |
| IIF8 | 6 | 1 |  |  |  |
| IIF9 | 6 | 1 |  |  |  |
| IIF10 | 5 | .83 | x |  | Word change from “occasionally” to “sometimes” |
| IIF11 | 6 | 1 |  |  |  |
| IIF12 | 6 | 1 |  |  |  |
| IIF13 | 5 | .83 | x |  | Phrasing of item changed |
| IIF14 | 5 | 0.83 |  |  |  |
| IIF15 | 5 | .83 | x |  | Suggested clarification from “plans to work” to “plans to work outside the home” |
| IIF16 | 5 | 0.83 |  |  |  |
| IIF17 | 5 | 0.83 | x |  | Word change from “restaurants” to “markets” as markets are seen as a more common public environment in this cultural context over restaurants. |
| IIF18 | 5 | 0.83 |  |  |  |
| IIF19 | 6 | 1 |  |  |  |
| IIF20 | 5 | 0.83 | x |  | Item expanded to include a broader range of mixed feeding options including, breast milk, formula, water, tea and porridge. |
| IIF21 | 6 | 1 |  |  |  |
| IIF22 | 6 | 1 |  |  |  |
| IIF23 | 6 | 1 |  |  |  |
| SN1 | 6 | 1 |  |  |  |
| SN2 | 6 | 1 |  |  |  |
| SN3 | 5 | 0.83 |  |  |  |
| SN4 | 5 | 0.83 |  |  |  |
| SN5 | 0 | 0 |  | x |  |
| SN6 | 6 | 1 |  |  |  |
| SN7 | 6 | 1 |  |  |  |
| BSE1 | 5 | .83 | x |  | Simplify word from “determine” to “I can tell” |
| BSE2 | 6 | 1 |  |  |  |
| BSE3 | 6 | 1 |  |  |  |
| BSE4 | 6 | 1 |  |  |  |
| BSE5 | 4 | 0.66 |  | x |  |
| BSE6 | 6 | 1 |  |  |  |
| BSE7 | 6 | 1 |  |  |  |
| BSE8 | 6 | 1 |  |  |  |
| BSE9 | 6 | 1 |  |  |  |
| BSE10 | 6 | 1 |  |  |  |
| BSE11 | 6 | 1 |  |  |  |
| BSE12 | 5 | 0.83 |  |  |  |
| BSE13 | 6 | 1 |  |  |  |
| BSE14 | 6 | 1 |  |  |  |
| BSE15 | 6 | 1 |  |  |  |
| BSE16 | 5 | 0.83 | x |  | Item simplified from identifying signs of “mastitis” to “breast health problems” |
| BSE17 | 6 | 1 |  |  |  |

*item codes and numbers match original instrument, prior to revision.

**IIF 18-23 are additional items created by study team representing “motivation” construct
